# Soluble CD14 Levels Reflect Liver Inflammation in Patients with Nonalcoholic Steatohepatitis

**DOI:** 10.1371/journal.pone.0065211

**Published:** 2013-06-07

**Authors:** Yuji Ogawa, Kento Imajo, Masato Yoneda, Takaomi Kessoku, Wataru Tomeno, Yoshiyasu Shinohara, Shingo Kato, Hironori Mawatari, Yuichi Nozaki, Koji Fujita, Hiroyuki Kirikoshi, Shin Maeda, Satoru Saito, Koichiro Wada, Atsushi Nakajima

**Affiliations:** 1 Department of Gastroenterology, Yokohama City University Graduate School of Medicine, Yokohama, Japan; 2 Department of Pharmacology, Osaka University Graduate School of Dentistry, Suita, Japan; The University of Hong Kong, Hong Kong

## Abstract

**Background & Aims:**

Liver inflammation is a risk factor for the progression of nonalcoholic fatty liver disease (NAFLD). However, the diagnosis of liver inflammation is very difficult and invasive liver biopsy is still the only method to reliably detect liver inflammation. We previously reported that overexpression of CD14 in Kupffer cells may trigger the progression to nonalcoholic steatohepatitis (NASH) via liver inflammation following hyper-reactivity to low-dose lipopolysaccharide. Therefore, the aim of this study was to investigate the relationship between soluble type of CD14 (sCD14) and histological features in patients with NAFLD.

**Methods:**

Our cohort consisted of 113 patients with liver biopsy-confirmed NAFLD and 21 age-matched healthy controls. Serum sCD14 levels were measured by an enzyme-linked immunosorbent assay.

**Results:**

Serum sCD14 levels were significantly associated with diagnosis of NASH and the area under the receiver operator characteristic curve (AUROC) to distinguish between not NASH and NASH was 0.802. Moreover, serum sCD14 levels were significantly associated with the disease activity based on NAFLD activity score and hepatic CD14 mRNA expression, which is correlated with membrane CD14 (mCD14) expression, in patients with NAFLD. In multiple regression analysis, the serum sCD14 levels were independently associated with liver inflammation. The AUROC to distinguish between mild and severe liver inflammation in patients with NAFLD was 0.752.

**Conclusions:**

We found that serum sCD14 levels increased significantly with increasing liver inflammation grade in patients with NAFLD, reflecting increased hepatic CD14 expression. Serum sCD14 is a promising tool to predict the worsening of liver inflammation, and may offer a potential biomarker for evaluation of therapeutic effects in NAFLD.

## Introduction

Nonalcoholic fatty liver disease (NAFLD) is a major cause of chronic liver injury in many countries [1], [2]. A recent study showed that the risk of developing NAFLD is 4–11 times higher in patients with metabolic syndrome, compared with healthy individuals [3]. NAFLD ranges from benign simple steatosis to nonalcoholic steatohepatitis (NASH), while NASH often progresses to severe fibrosis [4], [5] and hepatocellular carcinoma [6]–[8]. In addition, the mechanisms involved in the development of NASH are not fully understood and the therapeutic options limited. Therefore, predicting the progression of simple steatosis to NASH and developing methods to facilitate the precise diagnosis of NASH are important targets for clinical research.

Inflammation is a central process in the pathogenesis of NASH. Previous reports have shown that chronic liver inflammation is an important contributing factor to the pathogenesis of NASH and the key predictor of histological progression [9]–[11]. Therefore, precise detection and evaluation of liver inflammation are important to help predict the progression of NASH. In fact, several clinical biomarkers associated with systemic inflammation, including serum high-sensitivity C-reactive protein (CRP) [12] and cytokines [13], have been proposed as potential markers of liver inflammation to aid NASH diagnosis. However, no clinical studies have confirmed the usefulness of these markers to date. Therefore, invasive liver biopsy is still the only method to reliably detect liver inflammation and reach a definite diagnosis of NASH. However, this procedure is invasive and is associated with a relatively high risk of complications [14], emphasizing the clinical importance of identifying biomarkers for liver inflammation in patients with NAFLD.

We recently discovered that leptin-induced overexpression of CD14 in the liver is an important component of the pathogenesis of NASH [15]. We found that CD14 overexpression resulted in a hyper-responsiveness to low-dose lipopolysaccharide (LPS), an important step in the progression from simple steatosis to steatohepatitis, and was associated with liver inflammation and fibrosis [15]. These results suggest that measuring hepatic CD14 expression, which reflects its expression in Kupffer cells, may be useful to predict liver inflammation in NASH. However, invasive biopsies are still required to collect the tissue samples used to measure liver CD14 expression.

CD14 is a co-receptor that is detected in two forms: a glycosylphosphatidylinositol-anchored membrane protein (mCD14) and a soluble serum protein (sCD14) lacking the anchor protein [16]. Additionally, several reports have shown that sCD14 is shed from the surface of mCD14-expressing cells [16]–[18], although the exact roles of sCD14 are still unknown. Therefore, we hypothesized that serum sCD14 levels, shed from mCD14, might be highly correlated with hepatic CD14 expression levels in NASH patients, and could predict the severity of NASH, particularly liver inflammation. If this hypothesis is correct, measuring serum sCD14 levels may be very useful to predict the progression of NASH and could become a routine test for the assessment in NAFLD patients for predicting NASH progression instead of invasive liver biopsy. Therefore, the purpose of this study was to investigate the clinical usefulness of measuring serum sCD14 levels as a biomarker for assessing the severity of NASH.

## Patients and Methods

### Subjects

The study population consisted of 113 patients with biopsy-confirmed NAFLD and 21 healthy control subjects, aged ≥20 years, who attended Yokohama City University between April 2007 and March 2012. We obtained written informed consent from all subjects before conducting examinations. The study was conformed to the ethical guidelines of the Declaration of Helsinki and approved by the Ethics Committee at Yokohama City University. Subjects with a history of excessive alcohol consumption (weekly consumption >140 g for men, >70 g for women), other liver diseases, use of drugs associated with fatty liver, and clinically significant weight loss, for example, were excluded. Twenty-one healthy subjects with a mean age and sex ratio similar to those of the NAFLD group were also enrolled. Liver enzyme levels and ultrasound scans were normal for all of the healthy subjects. For the purpose of this study, subjects diagnosed with diabetes mellitus before the present admission and subjects with fasting plasma glucose >126 mg/dl and/or serum HbA1c >6.1% were defined as having diabetes mellitus. Subjects taking antidyslipidemic drugs and subjects with cholesterol >220 mg/dl and/or triglyceride >150 mg/dl were defined as having dyslipidemia. Subjects using antihypertensive drugs and subjects with resting blood pressure exceeding 130/85 mmHg on at least two occasions were defined as having hypertension.

### Clinical and Laboratory Evaluations

Body weight and height were measured with a calibrated scale after the subjects had removed their shoes and any heavy clothing. Venous blood samples were obtained after an overnight (12 h) fast and were used to measure serum glucose, aspartate aminotransferase (AST), alanine aminotransferase (ALT), CRP, ferritin, and insulin. Serum insulin levels were measured using a radioimmunoassay. The other parameters were measured using a conventional automated analyzer. Insulin resistance was calculated using the modified homeostasis model assessment of insulin resistance (HOMA-IR) equation: HOMA-IR = fasting insulin (µU/ml) × plasma glucose (mg/dl)/405, as originally reported by Matthews et al [19].

### Anthropometry and Abdominal Fat Distribution

Abdominal fat distribution was determined by computed tomography (CT) with the subjects in a supine position, as previously described [20]. Subcutaneous fat area and intra-abdominal visceral fat area were measured at the level of the umbilicus using a standardized method based on CT values. A histogram representing the fat tissue was computed based on the mean attenuation ±2 standard deviations (SDs).

### Measurement of Serum sCD14 Levels

Serum sCD14 levels were measured by a sandwich enzyme-linked immunosorbent assay (R&D Systems, Abingdon, UK). The intra- and interassay coefficients of variation stated by the manufacturers were <7% for all assays.

### Histological Assessment

We performed liver biopsies with an 18G needle biopsy kit using a standard protocol. Two specimens were obtained to provide a sufficient sample size for analysis and to reduce histological errors. Liver biopsy samples were excised and embedded in paraffin for histological analysis. The presence of collagen, as an index of lesion fibrosis, was examined in Masson’s trichrome-stained preparations. Histological assessment of liver was performed by two pathologists according to criteria proposed by Sanyal and Brunt et al. [21]–[22]. Macrovesicular steatosis affecting at least 5% of the hepatocytes was observed in all the cases of NAFLD, and the patients were classified as not having steatohepatitis (not NASH) or having steatohepatitis (NASH). In addition to steatosis, the minimum criteria for the diagnosis of steatohepatitis include the presence of lobular inflammation, ballooning of cells and perisinusoidal/pericellular fibrosis in zone 3 of the hepatic acini. Steatosis was graded as <5% (grade 0), 5–33% (grade 1), 33–66% (grade 2), and >66% (grade 3). Lobular inflammation was graded according to the number of inflammatory foci per field of view at a magnification of 200×, as follows: no foci = 0, <2 foci per field = 1, 2–4 foci per field = 2, and >4 foci per field = 3. Hepatocellular ballooning was graded as none (grade 0), few ballooning cells (grade 1), and many balloon cells (grade 2). The pathologists also performed NAFLD activity scoring based on Kleiner et al [23] to evaluate disease activity, but the actual diagnosis was not based on NAFLD activity score (NAS) [21]. The NAS was calculated as the unweighted sum of the scores for steatosis, lobular inflammation, and hepatocellular ballooning based on the Nonalcoholic Steatohepatitis Clinical Research Network methodology. The severity of fibrosis was scored according to the method of Brunt [22]. Subjects with NASH-associated cirrhosis were defined according to a previously proposed clinicopathological classification [24].

### RNA Isolation and Real-Time PCR Analysis

Total RNA was extracted from liver tissue samples from patients with NAFLD (n = 70) using the RNeasy mini kit (QIAGEN, Tokyo, Japan). The mRNA expression levels of human CD14 and β-actin were determined in liver tissue by fluorescence-based RT-PCR on an ABI PRISM 7700 Sequence Detection System (Life Technologies, Carlsbad, CA).

### Cell Culture

The murine monocyte/macrophage cell line RAW264.7 was obtained from ATCC (Rockville, MD). Cells were cultured at 37°C under 5% CO2 in Dulbecco’s modified Eagle’s medium (ASAHI TECHNO GLASS Co., Tokyo, Japan), and supplemented with 100 units/mL penicillin and 100 mg/mL streptomycin plus 10% fetal bovine serum. After incubation, the medium was treated with LPS (10 ng/mL) in PBS for 2 or 4 h. PBS supernatants were recovered, treated with protease inhibitor mixture (Sigma-Aldrich), and centrifuged at 10,000 x *g* for 10 min, following the analysis of sCD14 in the culture medium using by a Western immunoblot analysis and a sandwich enzyme-linked immunosorbent assay. Proteins were incubated with anti-mouse CD14 antibodies (BD Pharmingen), and HRP-conjugated secondary antibody (Cell Signaling Technology).

### Statistical Analysis

Continuous variables are summarized as means ± standard deviation, while categorical variables are summarized as percentages. Spearman's correlation coefficient was used to determine the correlations between serum sCD14 levels and the factors of interest. The *t*-test was used for univariate comparisons between groups of subjects. Because many of the variables were not normally distributed, we used the Kruskal–Wallis test for comparisons of more than two independent groups. We assessed the diagnostic performance of serum sCD14 levels by analyzing the receiver operating characteristic (ROC) curves. The ROC curve is a plot of sensitivity versus 1– specificity for all possible cutoff values. The most commonly used index of accuracy is the area under the ROC curve (AUROC), with values close to 1.0 indicating a high diagnostic accuracy. The accuracy of serum sCD14 levels for discriminating between mild and severe inflammation was determined by calculating the sensitivity, specificity, positive predictive value (PPV), and negative predictive value (NPV). Multivariate analysis was performed using logistic regression analysis. In all analyses, values of *P*<0.05 were considered statistically significant. All statistical analyses were performed using SPSS software version 12.0 (SPSS, Inc., Chicago, IL).

## Results

### Patient Characteristics

The clinical and biochemical characteristics of the healthy controls (n = 21), and patients without NASH (n = 48), and NASH (n = 65) are shown in Table 1. The histological findings of the liver biopsy specimens taken from the patients with NAFLD are shown in the lower part of Table 1. The inter-observer agreement between the two pathologists for liver fibrosis stage was 90.2%.

**Table 1:** Clinical and serological characteristics of the control and patient population.

	Controls	Not NASH	NASH	P value*
Number (n)	21	48	65	
Age (years)	44.8±9.0	50.4±13.6	51.4±12.8	0.381
Gender (male; female)	14;7	26;22	36;29	0.328
Body mass index (kg/m^2^)	21.9±2.6	27.9±5.3	29.1±5.1	0.046
Visceral fat area (cm^2^)		141.1±34.9	151.4±40.4	0.171
Subcutaneous fat area (cm^2^)		191.3±54.1	201.9±58.1	0.173
Fasting blood sugar (mg/dl)	84.2±10.1	101.2±25.1	106.2±29.4	0.301
AST (IU/l)	23.8±4.8	40.1±16.1	41.5±17.1	0.284
ALT (IU/l)	22.8±6.2	49.3±27.2	54.3±26.9	0.212
C-reactive protein (mg/l)	0.27±0.21	0.73±0.47	1.35±0.94	0.011
HOMA-IR	0.96±0.17	2.61±1.39	3.66±2.01	0.009
Dyslipidemia (%)	0	17 (35.1)	23 (47.9)	0.052
Hypertension (%)	0	19 (39.5)	20 (41.5)	0.522
Steatosis grade				0.003
5–33%		22	23	
33–66%		19	31	
>66%		7	11	
Lobular inflammation				7 x 10^−12^
None		15	0	
<2 foci per 200x field		23	31	
2–4 foci per 200x field		8	22	
>4 foci per 200x field		2	12	
Liver cell ballooning				3 x 10^−16^
None		30	0	
Few balloon cells		16	47	
Many balloon cells		2	18	
Fibrosis stage				3 x 10^−16^
None		21	0	
Perisinusoidal or periportal		23	38	
Perisinusoidal and portal/periportal		4	20	
Bridging fibrosis		0	5	
Cirrhosis		0	2	

Numbers represent the mean ± SD. Abbreviations: AST, aspartate aminotransferase; ALT, alanine aminotransferase; HOMA-IR, homeostasis model for the assessment of insulin resistance. P values correspond to the comparison of the three subjects groups (not NASH, borderline NASH and definite NASH) using the Kruskal–Wallis tests for continuous factors.

### Serum sCD14 Levels

**Figure 1:**
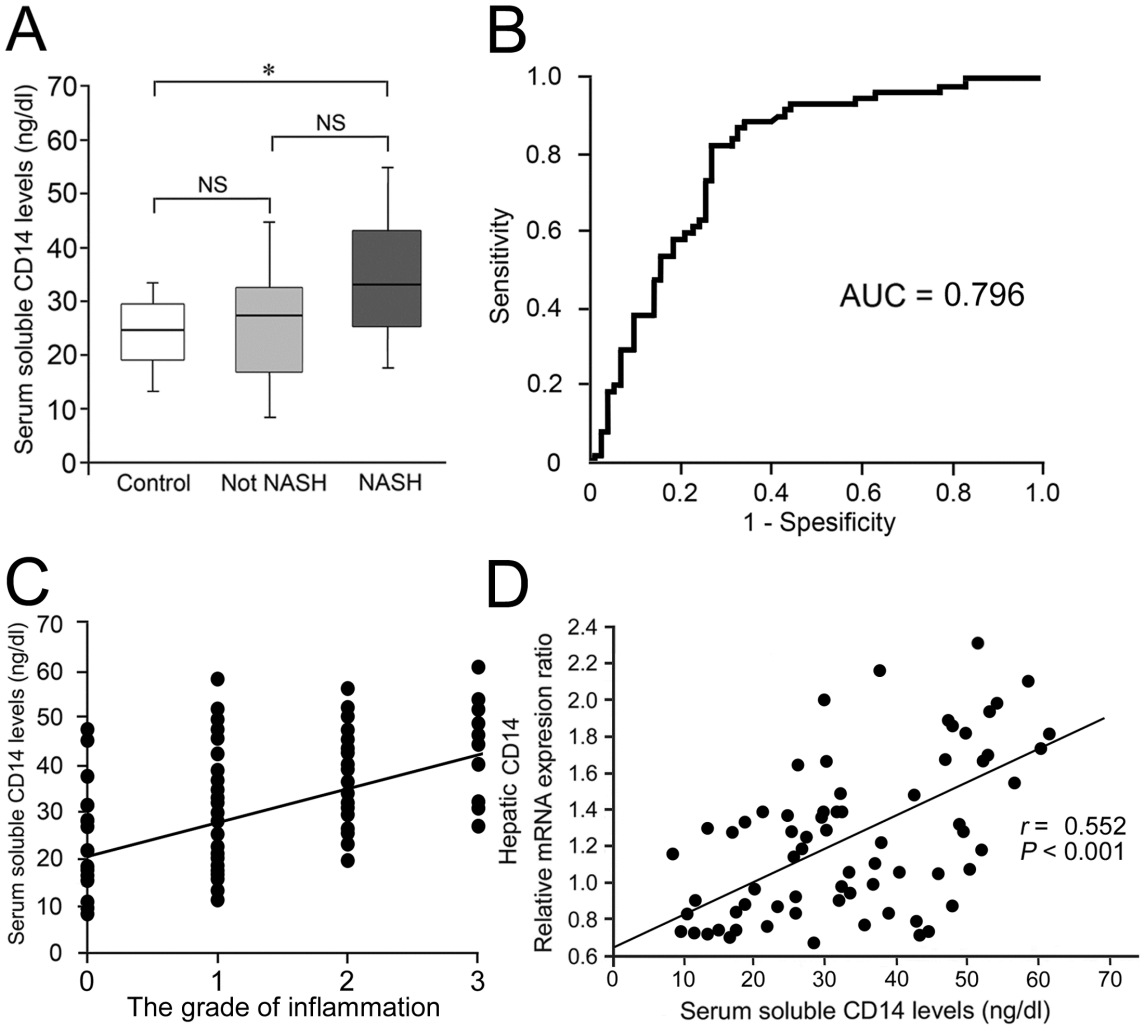
Serum sCD14 levels, liver inflammation and hepatic CD14 expression. (A) Serum sCD14 levels in control subjects and patients with NAFLD. The graph shows the interquartile range (box), median (the line), and range (lines) of serum sCD14 levels. The median (range) values (ng/dl) are 24.3 (13.3–32.4), 27.5 (9.21–44.2), and 32.2 (17.9–54.2) for control subjects (*n* = 21), not NASH (*n* = 48), and NASH (*n* = 65), respectively. Statistical significance was determined by analysis of variance with Scheffe’s correction for multiple testing. *P<0.05. (B) Receiver operating characteristic (ROC) curve and area under the ROC curve (AUROC) for distinguishing between not NASH (n = 48) including control subjects (n = 21) and NASH (n = 65) using serum sCD14 level. (C) Relationship between serum sCD14 and the grade of liver inflammation in patients with NAFLD. Serum sCD14 levels are significantly correlated with the grade of liver inflammation (Spearman’s *r* = 0.498, *P*<0.001). (D) Relationship between serum sCD14 and hepatic CD14 mRNA expression in patients with NAFLD. Serum sCD14 levels are significantly correlated with hepatic CD14 mRNA expression levels (Spearman’s *r* = 0.552, *P*<0.001). The correlation was determined in 69 patients with NAFLD patients.

We measured serum sCD14 levels in the healthy controls and in the two subgroups of patients with NAFLD. We found no marked differences in the serum CD14 levels between the healthy controls and patients without NASH (Fig. 1A). However, the serum sCD14 level was markedly elevated in patients with NASH compared with that in patients without NASH (Fig. 1A). The area under the receiver operating characteristic (ROC) curve for distinguishing between not NASH and NASH using serum sCD14 level was 0.796 (Fig. 1B). Using a cutoff level of greater than 27.3 ng/ml for serum sCD14 level yielded sensitivity and specificity values of 81.5% and 72.5%, respectively. The positive and negative predictive values for the serum sCD14 level of 27.3 ng/ml were 73.6% and 80.6%, respectively.

The correlations between serum sCD14 levels and the clinical characteristics of patients with NAFLD are shown in Table 2. Serum sCD14 levels were significantly correlated with NAS ( Spearman’s *r* = 0.354, *P* = 0.004) and with individual NAS components, including lobular inflammation, ballooning, and fibrosis. The strongest positive correlation was observed between the serum sCD14 level and the grade of lobular inflammation (*r* = 0.498, *P*<0.001) (Table 2 and Fig. 1C). These results indicate that serum sCD14 levels closely reflect the disease activity of NAFLD, especially the grade of liver inflammation.

**Table 2:** Correlations between serum sCD14 level and clinical parameters.

Factor	rho	P-value
Age (years)	−0.082	0.871
Body mass index (kg/m^2^)	0.021	0.522
Visceral fat area (cm^2^)	0.123	0.354
Subcutaneous fat area (cm^2^)	−0.053	0.517
Fasting Blood Sugar (mg/dl)	0.105	0.188
AST (IU/l)	0.136	0.153
ALT (IU/l)	0.214	0.049
C-reactive protein (mg/l)	0.223	0.047
HOMA-IR	0.217	0.052
NAS	0.354	0.004
Steatosis	−0.042	0.492
Inflammation	0.498	<0.001
Ballooning	0.274	0.051
Fibrosis	0.365	<0.001

Numbers represent the mean ± SD. Abbreviations: AST, aspartate aminotransferase; ALT, alanine aminotransferase; HOMA-IR, homeostasis model for the assessment of insulin resistance; NAS, NAFLD activity score. The correlation between serum sCD14 levels and other parameters is examined by Spearman correlations coefficient.

Next, we investigated the relationship between serum sCD14 levels and hepatic CD14 mRNA expression using liver biopsies from patients with NAFLD. As shown in Fig. 1D, the serum sCD14 levels were significantly correlated with the hepatic CD14 mRNA expression levels in patients with NAFLD (*r* = 0.552, *P*<0.001). These results indicate that serum sCD14 levels in patients with NAFLD closely reflect the hepatic CD14 expression levels.

### Multiple Regression Analysis to Predict Lobular Inflammation in Patients with NAFLD

Next, we investigated the correlation between serum sCD14 levels and other clinical factors that may be involved in lobular inflammation in NAFLD using multiple regression analysis. For this analysis, we divided patients with NAFLD into two categories, mild inflammation (grade 0–1) and severe inflammation (grade 2–3), according to the criteria. The clinical and biochemical characteristics of both groups of patients are shown in Table 3. We performed multiple logistic regression analysis using age, BMI, ALT, CRP, and sCD14, which were significantly higher in patients with severe liver inflammation compared with patients with mild inflammation in univariate analyses. We also included sex in the multivariate analysis because the sex ratio was also significantly different between the two groups. Notably, only the serum sCD14 level was independently associated with grade of liver inflammation in the multiple regression analysis (Table 4).

**Table 3:** Clinical and serological characteristics of NAFLD patients with mild and severe liver inflammation.

	Grade 0–1 liver inflammation	Grade 2–3 liver inflammation	P value*
Number (n)	43	70	
Age (years)	47.2±13.2	52.3±12.9	0.046
Gender (male; female)	23;20	43;27	0.037
Body mass index (kg/m^2^)	27.9±5.3	29.9±5.9	0.042
Visceral fat area (cm^2^)	140.7±35.1	149.8±46.2	0.051
Subcutaneous fat area (cm^2^)	199.5±44.9	191.9±48.1	0.226
Fasting Blood Sugar (mg/dl)	105.2±13.1	110.2±13.4	0.251
AST (IU/l)	42.3±14.1	43.2±14.3	0.430
ALT (IU/l)	45.5±12.9	57.1±17.6	0.048
C-reactive protein (mg/l)	0.73±0.46	1.18±0.98	0.043
HOMA-IR	3.43±1.33	3.59±1.31	0.431
sCD14 (ng/dl)	25.7±10.5	31.2±11.6	0.009

Numbers represent the mean ± SD. Abbreviations: AST, aspartate aminotransferase; ALT, alanine aminotransferase; HOMA-IR, homeostasis model for the assessment of insulin resistance. P values correspond to the comparison between grade 0–1 liver inflammation and grade 2–3 liver inflammation in NAFLD patients using the Student’s t-test for continuous factors.

**Table 4:** Multiple logistic regression analysis of factors associated with grade 2–3 liver inflammation compared to grade 0–1 liver inflammation in NAFLD patients.

Factor	Odds ratio	95% CI	P value
Age (years)	1.071	0.992–1.149	0.0729
Gender	1.976	0.241–17.49	0.5287
Body mass index(kg/m^2^)	1.110	0.881–1.329	0.3987
ALT (IU/l)	0.995	0.938–1.029	0.2756
C-reactive protein(mg/l)	1.395	0.827–2.339	0.2131
sCD14 (ng/dl)	8.853	1.221–63.08	0.0116*

Abbreviations: ALT, alanine aminotransferase; sCD14, soluble CD14.

### Receiver Operator Characteristic Curve for liver inflammation

**Figure 2:**
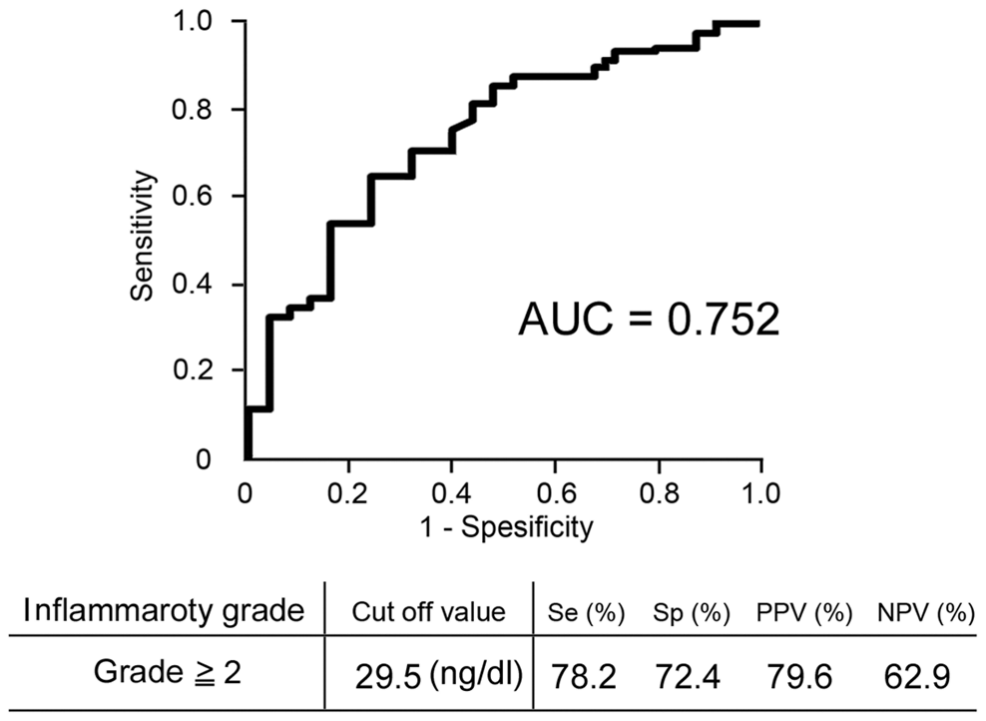
Serum sCD14 levels for diagnosis of the grade of liver inflammation. Receiver operating characteristic (ROC) curve and area under the ROC curve (AUROC) for discriminating between patients with severe (grade 2–3) or mild (grade 0–1) liver inflammation using serum sCD14 levels in 113 patients are shown. Serum sCD14 levels can diagnose severe liver inflammation in patients with NAFLD with moderate accuracy.

To discriminate between severe (grade 2–3) and mild (grade 0–1) liver inflammation in NAFLD, we plotted ROC curves and calculated the AUROC (Fig. 2). The resulting AUROC was 0.752. Based on the ROC curve, the optimal cutoff level for severe liver inflammation was 29.5 ng/dl. The sensitivity, specificity, PPV, and NPV were 78.2, 72.4, 79.6, and 62.9%, respectively.

### LPS-induced sCD14 *in vitro*

**Figure 3:**
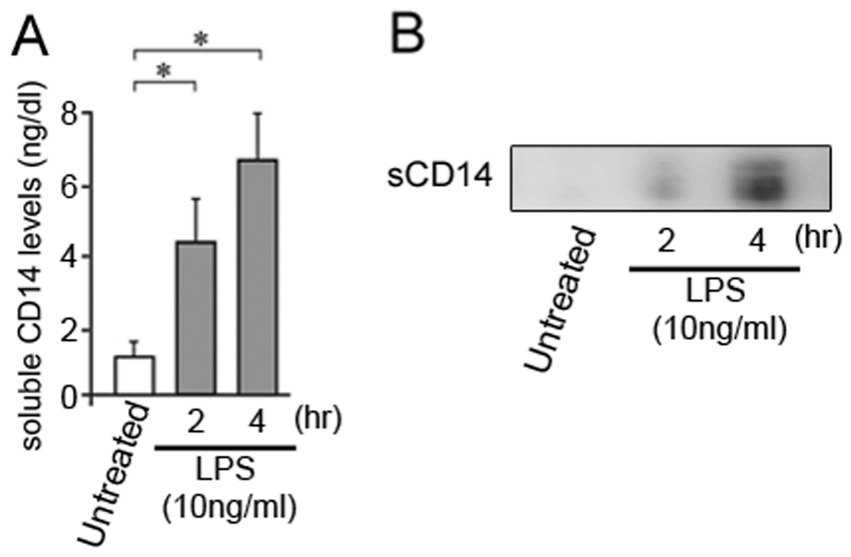
Lipopolysaccharide (LPS) increases sCD14 *in vitro*. sCD14 in cell culture medium from sham- and LPS-treated RAW264.7 cells was compared by (A) Western immunoblot analysis and (B) a sandwich enzyme-linked immunosorbent assay. LPS increased sCD14 in cell culture medium from RAW 264.7 cells. The immunoblot is representative of three independent experiments. Results are presented as means ± SD. Statistical significance was determined using ANOVA with Scheffe’s multiple testing correction (*p value <0.05).

Finally, to elucidate whether LPS can directly affect secretion of sCD14 from macrophages, we investigated sCD14 levels in the culture medium of RAW264.7 cells, the murine monocyte/macrophage cell line, incubated with LPS using by Western immunoblot analysis and a sandwich enzyme-linked immunosorbent assay. LPS treatment for RAW 264.7 cells significantly increased sCD14 in the cell culture medium (Fig. 3). These results support the observation that LPS increases secretion of sCD14 from macrophages including Kupffer cells.

## Discussion

In the present study, we showed that serum sCD14 levels were significantly associated with diagnosis of NASH. Moreover, the increased sCD14 levels in NASH patients were highly correlated with increased hepatic CD14 expression and liver inflammation, even after adjusting for age, sex, presence of diabetes, dyslipidemia, hypertension, BMI, VFA, and SFA. To our knowledge, this is the first report to show that sCD14 in blood is a promising biomarker for diagnosis of NASH and assessing liver inflammation in NASH.

NAFLD, which can progress to steatohepatitis and cirrhosis, is perhaps the most common type of chronic liver disease in obese patients [4]–[5]. As effective therapies for NAFLD have not yet been established, the identification of risk factors for disease progression, such as severe liver inflammation, would help to guide the implementation of risk-reduction strategies for these patients [25]. However, discrimination between mild and severe liver inflammation in patients with NASH is very difficult if using imaging modalities alone [26]. Indeed, liver biopsy examination is currently the only method that can precisely diagnose NASH and evaluate liver inflammation in patients with NASH [27]. However, liver biopsy is invasive, expensive, and is associated with a relatively high risk of complications [14]. Moreover, the accuracy of the procedure used to assess the severity of liver fibrosis is questionable because of intra- and inter-observer variation [27]–[31]. Sampling error has also been reported, even in patients with NASH [32]. Therefore, a non-invasive, reproducible, conceptually simple, and highly reliable test is needed to diagnose NASH and evaluate the severity of liver inflammation in patients with NASH.

CD14 is an effective mediator for the activation of monocytes in response to bacterial endotoxins. The serum sCD14 levels increase during the systemic response to bacterial invasion and endotoxin. Actually, we showed that sCD14 was increased in the culture medium of RAW264.7 cells after LPS treatment, suggesting that sCD14 may be shed from mCD14 in RAW264.7 cells under the effect of LPS. Here, we presented a hypothesis that the increased sCD14 levels in patients with NASH might reflect the severity of liver inflammation. Consistent with this hypothesis, we observed as significant association between serum sCD14 levels and definite NASH or the grade of liver inflammation in histological sections in liver biopsy-confirmed NAFLD. Furthermore, our previous report showed that leptin-induced upregulation of hepatic CD14 and the resulting hyper-reactivity to low-dose LPS during NASH progression were closely associated with increased liver inflammation [15]. These results were confirmed by the observation that hepatic CD14 expression was much higher in patients with NASH than in healthy controls and patients with nonalcoholic fatty liver [15]. In the present study, serum sCD14 levels were positively correlated with hepatic CD14 expression levels in patients with NAFLD. These results suggest that serum sCD14 levels might increase following increasing liver inflammation in NAFLD, reflecting increased hepatic CD14 expression. In other words, the sCD14 is likely to be liver CD14 that is shed into the blood.

Determination of the severity of liver inflammation is important to evaluate the prognosis of patients with NASH. To explore the clinical usefulness of sCD14 as a biomarker for liver inflammation, we investigated the diagnostic ability of serum sCD14 levels using multiple regression analysis and ROC curves. We found that serum sCD14 levels are independently associated with increased risk of severe liver inflammation in NAFLD patients. In addition, we found that a serum sCD14 cutoff level of 29.5 ng/dl showed good sensitivity and specificity for liver inflammation in patients with NAFLD, with values of 78.2 and 72.4%. The resulting AUROC was 0.752, indicating moderate accuracy. These results indicate that the serum sCD14 level is a good biomarker for liver inflammation in patients with NAFLD. A previous report showed that serum sCD14 levels in patients with NASH increased with increasing fibrosis stage; however, that report did not evaluate liver inflammation [33]. By contrast, we showed that serum sCD14 levels are strongly correlated with the grade of liver inflammation but not the stage of liver fibrosis. The serum sCD14 levels were also positively correlated with hepatic CD14 expression levels in patients with NAFLD. These results suggest that increased serum sCD14 levels reflect liver inflammation in NAFLD patients. Similarly, previous report showed that circulating microparticles from CD14 positive cells were correlated with severity of liver inflammation in patients with NAFLD [34]. However, we believe that sCD14 is a very convenient tool for evaluation of liver inflammation grade when compared with microparticles.

Several limitations of our study should be discussed. First, we did not conduct liver biopsies in the healthy control group for ethical reasons. Second, some patient selection bias may exist because liver biopsy may have been reserved for patients with NAFLD who were deemed likely to have NASH. Third, using liver biopsy as the ‘gold standard’ for assessing the accuracy of sCD14 has important limitations associated with sampling errors, as well as intra- and inter-observer variability, which are at least partly linked to the biopsy size [32]. Finally, serum sCD14 levels may increase in other conditions such as cholestasis, biliary atresia, and ischemia reperfusion injury [35]–[36]. However, these are extremely unusual conditions.

In conclusion, we confirmed that serum sCD14 may be a useful and non-invasive biomarker for diagnosis of NASH and assessing liver inflammation in patients with NAFLD, who are at high risk of progressing to advanced liver fibrosis. Further research, including larger-scale clinical studies or combination of serum sCD14 and other non-invasive biomarkers of NASH such as CK18, are needed to fully investigate the diagnostic and therapeutic implications of our findings.
